# The Impact of Space Flight on Survival and Interaction of *Cupriavidus metallidurans* CH34 with Basalt, a Volcanic Moon Analog Rock

**DOI:** 10.3389/fmicb.2017.00671

**Published:** 2017-04-28

**Authors:** Bo Byloos, Ilse Coninx, Olivier Van Hoey, Charles Cockell, Natasha Nicholson, Vyacheslav Ilyin, Rob Van Houdt, Nico Boon, Natalie Leys

**Affiliations:** ^1^Microbiology Unit, Belgian Nuclear Research Centre, SCK•CENMol, Belgium; ^2^Center for Microbial Ecology and Technology, Ghent UniversityGhent, Belgium; ^3^Research in Dosimetric Applications, Belgian Nuclear Research Centre, SCK•CENMol, Belgium; ^4^UK Centre for Astrobiology, School of Physics and Astronomy, University of EdinburghEdinburgh, UK; ^5^Institute of Medical and Biological Problems of Russian Academy of SciencesMoscow, Russia

**Keywords:** microbe-mineral interactions, space flight, *Cupriavidus metallidurans* CH34, basalt, FOTON

## Abstract

Microbe-mineral interactions have become of interest for space exploration as microorganisms could be used to biomine from extra-terrestrial material and extract elements useful as micronutrients in life support systems. This research aimed to identify the impact of space flight on the long-term survival of *Cupriavidus metallidurans* CH34 in mineral water and the interaction with basalt, a lunar-type rock in preparation for the ESA spaceflight experiment, BIOROCK. Therefore, *C. metallidurans* CH34 cells were suspended in mineral water supplemented with or without crushed basalt and send for 3 months on board the Russian FOTON-M4 capsule. Long-term storage had a significant impact on cell physiology and energy status (by flow cytometry analysis, plate count and intracellular ATP measurements) as 60% of cells stored on ground lost their cell membrane potential, only 17% were still active, average ATP levels per cell were significantly lower and cultivability dropped to 1%. The cells stored in the presence of basalt and exposed to space flight conditions during storage however showed less dramatic changes in physiology, with only 16% of the cells lost their cell membrane potential and 24% were still active, leading to a higher cultivability (50%) and indicating a general positive effect of basalt and space flight on survival. Microbe-mineral interactions and biofilm formation was altered by spaceflight as less biofilm was formed on the basalt during flight conditions. Leaching from basalt also changed (measured with ICP-OES), showing that cells release more copper from basalt and the presence of cells also impacted iron and magnesium concentration irrespective of the presence of basalt. The flight conditions thus could counteract some of the detrimental effects observed after the 3 month storage conditions.

## Introduction

Microorganisms can interact with rocks and minerals to enhance leaching of elements for sustaining their survival and growth. They can impact rock and mineral weathering through production of organic acids and other ligands, which in turn impact mineral solubility, denudation, and speciation (Dong, 2010). These microbe-mineral interactions are in fact essential for soil formation through biotransformation, biochemical cycling, and bioweathering (Gadd, 2010). In addition, they can be useful for and have already been applied in industry. For example, acidophilic iron- and sulfur oxidizing bacteria are used in bio-mining applications to oxidize copper and gold sulfidic bonds in order to solubilize and recover the economically interesting metals from the ores (Ubaldini et al., 2000). These interactions can also lead to the formation of biofilm communities on the mineral surface, in which members will be protected from harsh environments (Harrison et al., 2005).

Microbe-mineral interactions have also become of interest for space exploration missions. At the moment, human presence in space needs to be fully supported from Earth. To reduce the costs and the dependency for supplies from Earth for future more distant space missions, current research is investigating if supplies can be generated from endogenous material on planets and asteroids, such as the regolith and rocks. Microorganisms can be used in this process of *in-situ* resource utilization (ISRU) to extract useful elements that could be applied as fertilizers in a life support system and in the formation of fertile soil for plant cultivation (Cockell, 2010).

Since space conditions have been shown to cause many changes in bacterial physiology, including changes in motility and biofilm formation (Brown et al., 2002; Leys et al., 2004; Horneck et al., 2010; Leroy et al., 2010; Kim et al., 2013), these conditions may also influence microbe-mineral interactions as microgravity eliminates mass-driven convection and only diffusion can impact element release and availability as well as alter microbe-mineral contact (Jánosi et al., 2002). To evaluate the possibility of microbe-based ISRU, the potential impact of space environmental conditions such as microgravity and radiation on microbe-mineral interactions need to be studied.

Our study aimed at investigating the influence of space conditions on these microbe-mineral interactions, by testing the impact of space flight conditions on the survival and biofilm formation of the bacterium *Cupriavidus metallidurans* CH34 in mineral water supplemented with basalt. *C. metallidurans* is a motile β-proteobacterium that is found in the natural communities of basaltic rock (Sato et al., 2004). Furthermore, the interaction of type strain CH34 with basalt has already been studied and indicated that stress and starvation responses are triggered in the presence of basalt (Bryce et al., 2016) and that strain CH34 can sequester iron from basalt to sustain its growth (Olsson-Francis et al., 2010). In addition, type strain CH34 has been used previously as test organism to investigate bacterial behavior in space (Leys et al., 2009).

Thus, in order to prepare for a potential future feasibility studies of the biomining process in space (the ESA BIOROCK experiment), here preliminary tests were performed to assess the impact of flight conditions on an inactive bacterial inoculum, and its interactions with basalt rock. Therefore, *C. metallidurans* CH34 was stored in mineral water supplemented with basaltic rock and send on board of the FOTON-M4 capsule for 3 months. After flight, cell survival, physiology, biofilm formation, and the elements leaching from basalt were investigated.

## Materials and methods

### Strain and media composition

*Cupriavidus metallidurans* type strain CH34 (Mergeay et al., 1985) was cultivated at 30°C on a shaker in dark, aerobic conditions in a Tris buffered mineral (284 MM) medium containing 2 g/l sodium gluconate (Merck) as sole and more selective carbon source (Mergeay et al., 1985). The composition of the mineral water (Chaudfontaine, Belgium) used for cell's suspensions is given in Table 1, according to manufacturer's analysis. Cultivable bacteria were enumerated as CFUs on R2A medium (2% agar; Thermo Scientific, Belgium) and 284 MM agar (2% agar). R2A medium is a general medium used to plate out environmental samples taken from drinking water or aqueous environments (Reasoner and Geldreich, 1985).

**Table 1:** **Composition of Chaudfontaine mineral water**.

**Composition**	**mg/l**
HCO_3_	305
Ca	65
Na	44
SO_4_	40
Cl	35
Mg	18
K	2.5
F	0.4
NO_3_	<0.1
pH	7.6
Dry weight (Total suspended solid; 180°C)	385

### Basalt composition

Basalt, an igneous volcanic rock, was used as analog to the basalt rock that is found on the Mare regions of the Moon which have a low Ti content (Anand et al., 2012). The basalt was taken from the mid-ocean ridge close to the Eyjafjallajökull volcano in Iceland. The composition of this basalt is given in Table 2.

**Table 2:** **Composition of basaltic rock used in the experiment**.

**%**		**ppm**	
SiO_2_	51.1	Nb	0.7
Al_2_O_3_	15.16	Zr	46.8
FeO	9.53	Y	25.1
MgO	8.87	Sr	64
CaO	12.36	Rb	0.5
Na_2_O	1.76	Zn	79.4
K_2_O	0.05	Cu	95.3
TiO_2_	0.91	Ni	131.6
MnO	0.18	Cr	351.1
P_2_O_5_	0.08	V	301.4
Sum	100	Ba	2.5
Lost on ignition (LOI)	−0.47	Sc	47.6

### Flight setup

Three independent cultures of *C. metallidurans* CH34 were grown to stationary phase (OD_600 nm_ ~ 1), cells were harvested and washed three times with 10 mM MgSO_4_ and re-suspended in mineral water (10^9^ cells/ml, OD_600 nm_ = 1). Five milliliters of this cell suspension was transferred to silicone cryogenic vials (VWR international, Belgium) and supplemented with or without 10 w/v % basalt, which was crushed to 1–2 mm in size, washed in deionized water and heat sterilized beforehand. A control without cells containing only water and basalt was also prepared. Two replicate sets were prepared: one for flight and one for ground control.

The prepared cryogenic vials were wrapped with parafilm to secure the caps. Active temperature loggers (Smartbuttons, ACR systems, Canada) were added to the packages as well as passive radiation sensors [Thermoluminescence detectors (TLDs) and optically stimulated luminescence detectors (OSLDs); (Goossens et al., 2006; Vanhavere et al., 2008)] to monitor the temperature changes and the total radiation dose cumulated over the duration of the experiment.

**Figure 1:**
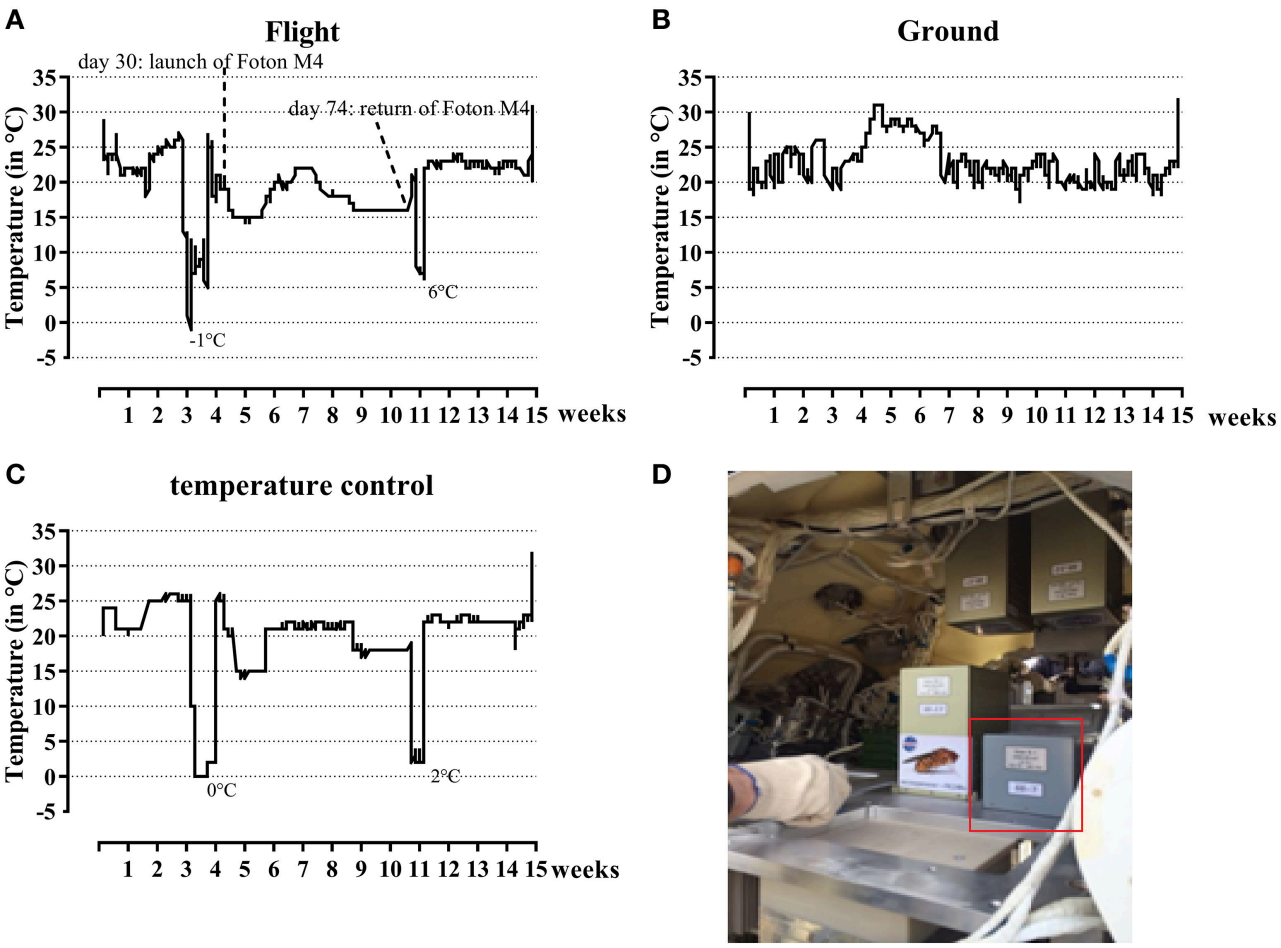
**Temperature profiles during the flight experiment and simulated control experiment and its setup in the FOTON capsule**. Both during flight **(A)** and ground **(B)** temperature profiles were determined. The temperature profile for the flight experiment was later on simulated **(C)** to determine the effect of these changes on the results. The experiment as set-up in the FOTON capsule is circled in red **(D)**.

The complete flight package was kept in the dark at ambient temperature (22.9 ± 1.8°C, Figure 1A) before and during transport from SCK•CEN (Mol, Belgium) to Moscow (Russia). It left SCK•CEN on 28th of June 2014, 3 weeks before the launch of the FOTON-M4 capsule. Ten days before launch, the samples were transported from Moscow (Russia) to the launch site (Baikonour, Kazakhstan), and put in the capsule 1 day before launch on July 18, 2014 (Figure 1D). Samples were kept at lower temperature during this transport (7.8 ± 3.9°C, Figure 1A). Inside the FOTON M4 capsule the experiment was kept at ambient temperature (17.6 ± 2.5°C, Figure 1A). The FOTON M4 capsule flew at 575 km altitude, with a 64.9° inclination in Low-Earth orbit and returned to Earth on September 1, 2014. Samples were returned from the landing site to Moscow at which temperatures were again low (7 ± 0.5°C). Samples then returned to SCK•CEN (Mol, Belgium) on September 30, 2014, while at ambient temperature (22.4 ± 2.1°C). The parallel ground control was kept at SCK•CEN at ambient temperature during the complete time period (22.9 ± 3°C, Figure 1B). Total radiation dose absorbed in water for the flight samples was measured and was 20.1 ± 1.47 mGy over the whole experiment duration. For the ground experiments the total dose was 1.2 ± 0.02 mGy.

To test the influence of temperature and storage conditions, a post-flight control ground experiment with and without basalt was performed. The cell suspensions and basalt were exactly prepared as mentioned in Section Flight Setup. Cryo tubes were placed in an incubator and the temperature profile of the flight experiment was simulated. At the end, the samples were analyzed exactly as described for the flight experiment (Section Post-flight Analysis). Data from this experiment is showed as “T ground” conditions in the results. The temperature profile is shown in Figure 1C. The data from these stimulated flight temperatures samples are used in the further results part of this paper.

### Post-flight analysis

After return to SCK•CEN, the samples were kept at ambient temperature and processed for analysis. Liquid was aspirated and 1 ml of this solution was transferred to sterile tubes for the following analyses: (1) two hundred microliters was used to estimate the number of viable cells by plating a serial dilution on R2A and 284 MM agar, (2) two hundred microliters was used for flow cytometry analysis, ATP and PHB measurements and (3) six hundred microliters was used to measure pH. The remaining 4 ml of the solution was used for ICP-OES analysis (see Section ICP-OES).

Contamination was revealed on the counting agar plates of the flight samples both on 284 MM and R2A in a level to 10^4^–10^5^cells/ml, as colonies visually different from CH34 were present until these dilutions. The cause of this contamination is unclear. No contaminations were found in the parallel ground control set, which was prepared and analyzed at the same time.

#### Sonication

After liquid aspiration, a piece of the basalt was aseptically removed from the tubes and analyzed with Scanning Electron Microscopy (see Section SEM). Next, 5 ml filtered mineral water was added and the solution was probe sonicated for 3 min at 20 kHz, 4 W at low-intensity to release the biofilm and intact cells from the biofilm from the basalt. The protocol for sonication was determined before the flight experiment based on previously described protocols (Kobayashi et al., 2009) and was found to be optimal at the conditions described above. After sonication, the solution was again analyzed as described in the post-flight analysis.

#### Flow cytometry

Samples were stained and analyzed with flow cytometry to analyze physiology and impact of space flight conditions on the CH34 cells. This was done according to the optimized procedures, described in Buysschaert et al. (2016), Van Nevel et al. and SLMB recommendation for characterizing drinking water communities (SLMB, 2012; Van Nevel et al., 2013; Buysschaert et al., 2016). Cell suspensions were diluted 10,000 times in 0.2 μm filtered Evian mineral water, as this gave the lowest background fluorescence. Next, the different dyes were added and cell suspensions were incubated at 35°C. The tested dyes include DiBAC_4_(3) (Sigma Aldrich, U.S.A.), cFDA (Sigma Aldrich, U.S.A.), SYBR Green (Sigma Aldrich), and PI (Sigma Aldrich). The different dye concentrations and incubations times are shown in Table 3. Work solutions were all prepared in DMSO and kept at 4°C. These were prepared from stock solutions in DMSO kept at −20°C. For cFDA and DiBAC_4_(3) cells were centrifuged and washed with Evian water before analyzing the samples with flow cytometry to eliminate background signals from the staining solution. For the other dyes, stained cell solutions were analyzed directly after incubation and the cells were not washed.

**Table 3:** **Used dyes for flow cytometer analysis, incubation times and concentration**.

**Stain**	**Analysis (<2,000 events/μl)**	**Incubation (35°C)**	**Final concentration**	**Excitation (nm)/Emission (nm)**
SYBR Green (SG)	All cells	13 min	1x	497/520
Propidium iodide (PI)	Permeabilized cells	13 min	0.2 μM	533/617
5(6)-Carboxyfluorescein diacetate (cFDA)	Active cells	20 min	10 μM	488/515
Bis(1,3-dibutylbarbituric acid) trimethine oxonol [DiBAC_4_(3)]	Membrane polarization and potential	20 min	5 μM	490/520

Stained bacterial suspensions were analyzed on an Accuri C6 (BD, Erembodegem) with a blue (488 nm, 20 mW) and red (640 nm, 14.7 mW) laser which was calibrated according to the manufacturer's recommendation. Standard optical filters were used and included FL-1 (530/30 nm), FL-2 (585/40 nm), and FL-3 (670 LP) for the blue laser and FL-4 (675/25 nm) for the red laser. The dyes DiBAC_4_(3), cFDA, and SYBR Green were all detected with FL-1, PI was detected on FL-3. A quality control with 6 and 8 peaks fluorescent beads (by manufacturer BD, Erembodegem) and cleaning cycle was performed prior to experiments to assess both the accuracy (bead count and position) and the cleanliness of the machine. Samples were analyzed using the Accuri C6 software (version 1.0.264.21). The flow files were uploaded to FlowCyte database (FR-FCM-ZYZQ).

The different dyes are used to test for different physiological parameters and determine the effect of the flight conditions on CH34. Sybr Green (SG) was used to stain all cells as this stain can enter both intact and damaged cells independent from their physiological state. SG enters the cells in both permeabilizated and non-permeabilizated cells due to its positive charge which allows it to pass it through the membrane (Veal et al., 2000; Berney et al., 2007, 2008). DiBAC_4_(3) staining allows differentiating between cells with an intact membrane potential and normal polarization and cells which have a depolarized membrane or lost membrane function as DiBAC_4_(3) can only enter the latter due to its anionic structure. Once in the cell it binds to positively charged proteins or hydrophobic regions. No binding of DiBAC_4_(3) with outer membrane structures is observed. Increased depolarization of the membrane also causes more influx of DiBAC_4_(3) while hyperpolarization causes a decrease in influx and thus fluorescence (Berney et al., 2008; Muller and Nebe-Von-Caron, 2010; Sträuber and Müller, 2010). cFDA staining can passively enter cells and is metabolized by esterase enzymes in the cytoplasm to its fluorescent product cFluorescein (cF) that accumulates in the cytoplasm and is thus an indicator for enzymatic cell activity. Negative charges present in cF ensures that it is better retained in the cell, avoiding leakage of the product as well as reducing background signal by unspecific binding (Sträuber and Müller, 2010).

#### SEM

To obtain an idea about cell morphology and biofilm structure Scanning Electron Microscopy (SEM) was performed. Five microliters of the solution as well as a piece of basalt was taken and transferred to a 0.2 μm filter (Milipore) and fixed two times with fixation solution [3% gluteraldehyde (w/v) in 0.15 M cacodylate solution, pH 7.6] in filter holders. Between each fixation step, the membrane was left to dry for 20 min at room temperature. Afterwards the filter surface was washed three times with the wash solution (0.15 M cacodylate solution). Next, filter holders were wrapped with parafilm and stored overnight at 4°C to let the filters dry. The next day, the filter surface was rinsed with ethanol, in ascending concentrations (30, 50, 70, 90, 95, 100% v/v), once for each concentration. Between each step the filters were left to dry for 10 min. The final rinsing with 100% ethanol was done three times. To dry the surface completely, the ethanol solution was replaced with hexamethyldisilazane (HMDS), and the filters were again rinsed three times, with 10 min of incubation between each step. Next, the filters were air-dried in a desiccator for storage. For visualization, these filters were taped onto an aluminum stub using carbon tape and coated with gold particles using the ScanCoat machine (2 × 300 ms, 6–8 mbar argon; 50 mA plasma tension). They were directly thereafter visualized with the JEOL JSM6610LV SEM Microscope with a W filament.

#### Intracellular ATP

To measure intracellular ATP levels the BIOTHEMA intracellular ATP kit HS was used and adapted from the manufacturer's protocol for smaller volumes. Cell suspensions were diluted 1/100 and 25 μl of this dilution was added in a cuvette. Twenty-five microliters of ATP eliminating agent was added and the cuvette was left to incubate for 10 min to degrade all the extracellular ATP. Twenty-five microliters of cell lysis “extractant BS” was added to the cell solution, vortexed and immediately thereafter 200 μl “ATP reagent HS” was added. Luminescence was measured immediately after (Kikkoman Lumitester c-100). Five microliters of a 100 nmol/l ATP standard was added as an internal control and light emission (luminescence) was measured again. From the overall amount of ATP (in pmol) measured in the samples, i.e., the value I, then the average intracellular ATP concentration per cell was calculated as follows:

$$\begin{array}{l} \frac{\left(\frac{I_{sample}}{I_{standard+sample}-I_{sample}}\right)}{total\;cell\;count\;\left(with\;SG\;in\;flow\right)\;in\;measured\;sample} \end{array}$$

#### Intracellular PHB

To measure intracellular PHB levels, a Nile Red staining protocol was developed, based on the work of Degelau et al. (1995), for *C. metallidurans* CH34 and adapted to our test conditions. Nile red binds selectively to non-polar lipid droplets inside cells and can be used to detect the presence of storage lipids (PHA/PHB) via fluorescence spectrophotometry (Greenspan and Fowler, 1985; Johnson and Spence, 2010). A Nile red working solution (200 mg/l) was prepared from a 1 g/l Nile red stock solution in DMSO, stored at −20°C. Ten microliters of the cell solution was diluted in 100 μl sterile Evian mineral water in a micro titer plate (MTP). Five microliters of the work solution was added and the MTP was incubated for 30 min at 30°C. Water without cells but with dye was used as a negative control and water without cells and without dye was used for background correction (blanco). Afterwards fluorescence of the lipid-bound Nile red was determined with a Thermo Scientific™ Fluoroskan Ascent™ Microplate Fluorometer. Nile red solution has an excitation peak at 544 nm and an emission peak at 590 nm. The normalized amount of intracellular PHB per cell was then calculated with following formula:

$$\begin{array}{l} \frac{\frac{\left(FI_{590\;nm,\;10\;\mu l\;sample}-FI_{590\;nm,\;negative\;control}-FI_{590\;nm,\;blanco}\right)}{OD_{600\;nm}}}{total\;cell\;count\;\left(with\;SG\right)\;in\;measured\;sample} \end{array}$$

#### ICP-OES

At the end of the experiment, the in-organic element concentrations in the water were measured to compare changes in water chemistry induced by the rocks and cells during the experiment. Four milliliters of the supernatant from the vials without basalt fragments was taken from the tubes and centrifuged (10,000 × g; 15 min, 20°C) to pellet the cells. This supernatant was then filtered through a 0.22 μm filter to remove particles and 20 μl of 70% nitric acid was added before final inorganic element concentrations in the cell-free supernatant were determined by ICP-OES (Inductively Coupled Plasma Optical Emission Spectroscopy). Details on the wavelengths used for ICP-OES analysis are provided in Supplementary Table S1. For concentrations below the detection limit, values were changed to zero and analyzed in the subsequent statistical analysis as such.

### Statistical analysis

For the statistical analysis of the data the GraphPad Prism (version 7.0) software package was used. For all the data of the cell suspension, normal distribution was assumed, while homogeneity of variances was tested with Levene's test and a two-tailed, one way ANOVA was used with Tukey *post-hoc* testing (alpha = 0.05). *P*-values of the statistical analysis are mentioned in Supplementary Table S2.

## Results

### The effect of space flight and basalt on cell survival and physiology

#### Effect of space flight on cultivability

**Figure 2:**
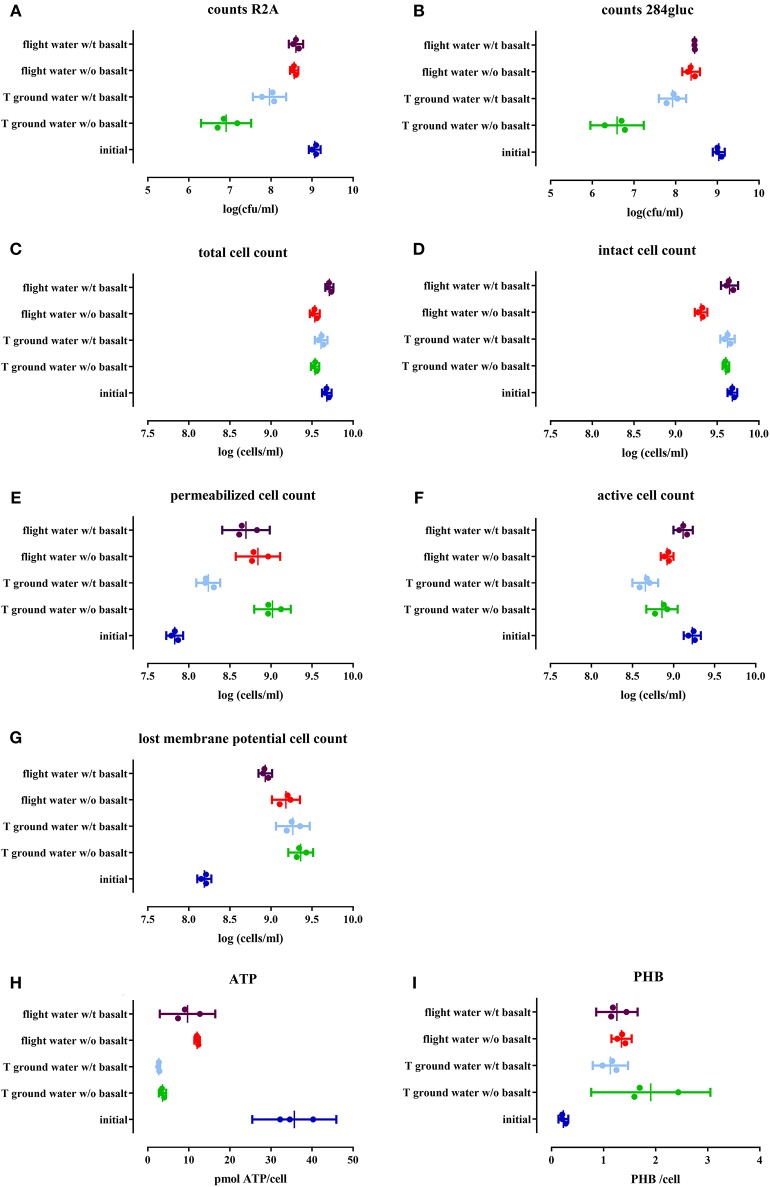
**Cell physiology, cultivability, ATP and PHB levels of an initial stationary phase culture of CH34 cells stored in mineral water with (indicated with w/t) and without (w/o) basalt, analyzed after the three month flight experiment and after the control temperature ground experiment**. All replicates are represented by a dot; the mean is indicated as a line as well as the 95% confidence interval between the brackets. For the statistical analysis, one way ANOVA was used with Tukey post testing (alpha = 0.05), *p*-values as well as significance are reported in Table 4 and in Supplementary Table S2. Samples were plated on R2A **(A)** and 284 MM **(B)** to determine cultivability of CH34. The total cells number (SG) **(C)** as well as the number of intact cells (SGPI) **(D)**, permeabilizated (SGPI) **(E)**, active (cFDA) **(F)**, and cells which have lost their membrane potential [DIBAC_4_(3)] **(G)** were measured. ATP **(H)** and PHB **(I)** content of the cells was also measured.

To assess cell viability and cultivability after space flight, a serial dilution of the cell suspensions was plated on R2A and 284 MM agar (Figures 2A,B respectively). Cultivability significantly decreased (*p* < 0.0001) in the space flight experiment, compared to the initial cell number, irrespective of the presence of basalt, leading to 50% of the population remaining cultivable. Statistical analysis have been summarized in Table 4. A greater loss of cultivability was observed for the ground experiment with only 1 and 0.1% of the population still being cultivable, with and without basalt, respectively. These results indicated that cultivability decreased significantly less during space flight compared to the ground control and that the presence of basalt positively affected cultivability of the ground control.

**Table 4:** **Summarizing table of the results for the different parameters of the planktonic cell fraction as well as the statistical significance (*P*-value), cultivability decrease and percentages of the population measured with flow cytometry**.

**Parameter**	**Condition**	**Amount**	**Significance**	***P*****-value**	**Amount**
Cultivability	Initial	9 log = 100%	Ground	<0.0001	95% log decrease
			Flight	0.0162	50% log decrease
	Flight		Ground	0.0025	90% decrease
	Basalt		Ground	<0.0001	90% log decrease
Total cell count	Initial		Ground	0.0336	
			Flight w/o basalt	0.0002	
	Flight		Ground	0.0185	
			Flight w/t basalt	<0.0001	
	Basalt		Ground	0.0247	
			Flight	<0.0001	
Intact	Initial	99%	Flight w/o basalt	<0.0001	60%
	Flight	73%	Flight w/o basalt	<0.0001	60%
	Basalt	86%	Flight w/o basalt	<0.0001	60%
Permeabilized	Initial	1%	Ground	0.0014	15%
			Flight	<0.0001	17%
	Flight	17%	Ground	0.0078	15%
			Flight w/t basalt	0.0006	10%
	Basalt	6%	Ground w/t basalt	<0.0001	4%
Active	Initial	35%	Ground	<0.0001	16%
			Flight w/o basalt	0.0003	24%
	Flight	24%	Ground	0.0013	16%
			Flight w/t basalt	<0.0001	25%
	Basalt	18%	Ground	0.0073	16%
			Flight	0.0095	25%
Lost membrane potential	Initial	3%	Ground	<0.0001	56%
			Flight	<0.0001	30%
	Flight	30%	Ground	0.0251	56%
			Flight w/t basalt	0.0003	17%
	Basalt	27%	Flight w/t basalt	0.0029	17%
ATP	Initial		Flight	<0.0001	
			Ground	<0.0001	
	Flight		Ground	0.0439	
PHB	Initial		Ground	0.005	
			Flight	0.002	
	Flight		Flight w/t basalt	0.0376	
	Basalt		Ground w/t basalt	0.0135	

#### Effect of space flight on physiology

The impact of space flight conditions and the presence of basalt on physiological changes within the cells were analyzed with flow cytometry by staining with specific functional dyes. The total cell numbers, measured with SYBR Green (SG), decreased slightly but significantly in samples without basalt (*p* < 0.0002) compared to the initial cell number as well as in the ground sample with basalt (*p* < 0.0336). When ground and flight samples were compared, flight samples with basalt significantly differed from the ground samples (*p* < 0.003, Figure 2C). When intact cell numbers were compared (by measuring SG positive cells when stained with both SG and PI, Figure 2D) to the initial setup (with 99% of the population intact in the initial setup) only the flight sample without basalt differs significantly, containing 60% intact cells (*p* < 0.0001). In addition, these samples also were significantly different from the flight samples with basalt (86%) (*p* < 0.0001) and ground with and without basalt (90%; *p* < 0.0001). Permeabilizated fractions (measuring the PI positive cells when stained with both SG and PI, Figure 2E) increased significantly in all test conditions (*p* < 0.0001) compared to the initial (contained 1% permeabilizated cells). Both flight conditions with and without basalt and ground control without basalt contained more permeabilizated cells then the ground with basalt, which only contained 5% permeabilizated cells. The number of cells stained with DIBAC_4_(3) (Figure 2F), related to cells that lost their membrane potential, significantly increased (*p* < 0.0014) in all conditions compared to the initial amount in the initial culture (3%). Less membrane potential-defected cells were observed in the flight samples with basalt (16%) significantly differing (*p* < 0.0078) from the ground samples and flight samples without basalt, indicating that the presence of basalt and flight conditions reduced the number of cells that lost their membrane potential. Cell activity measurements, with cFDA (Figure 2G) showed that the number of active cells in the flight experiment with basalt did not significantly differ (*p* > 0.1647) from the initial setup (35%), in contrast to the other samples (*p* < 0.0095) with decreased numbers of active cells. Basalt also increased the number of active cells in the flight sample with basalt (26%) compared to other conditions.

#### Effect of space flight on energy status

Intracellular ATP was measured to determine the energy status of the cells (Figure 2H). When ATP levels were compared with the initial culture (*p* < < 0.0001), ATP levels decreased 3-fold in the stored cells in water, in all conditions. Flight had a significant effect (*p* < 0.0439) on ATP levels with flight samples containing more ATP/cell then the ground samples. Basalt did not impact ATP levels, neither for ground nor flight samples. In addition, the energy stock of the cells was estimated by measuring PHB content (Figure 2I). The PHB content per cell significantly increased after the 3 months of storage of the cells in water (*p* < 0.005), in all conditions, compared to the initial culture and on average doubling PHB concentration. No significant difference (*p* > 0.0792) was seen between ground and flight samples except for the ground samples without basalt (*p* < 0.0376) which contained more PHB then the ground and flight samples with basalt.

### The effect of basalt and space flight on biofilm formation

#### SEM microscopy

**Figure 3:**
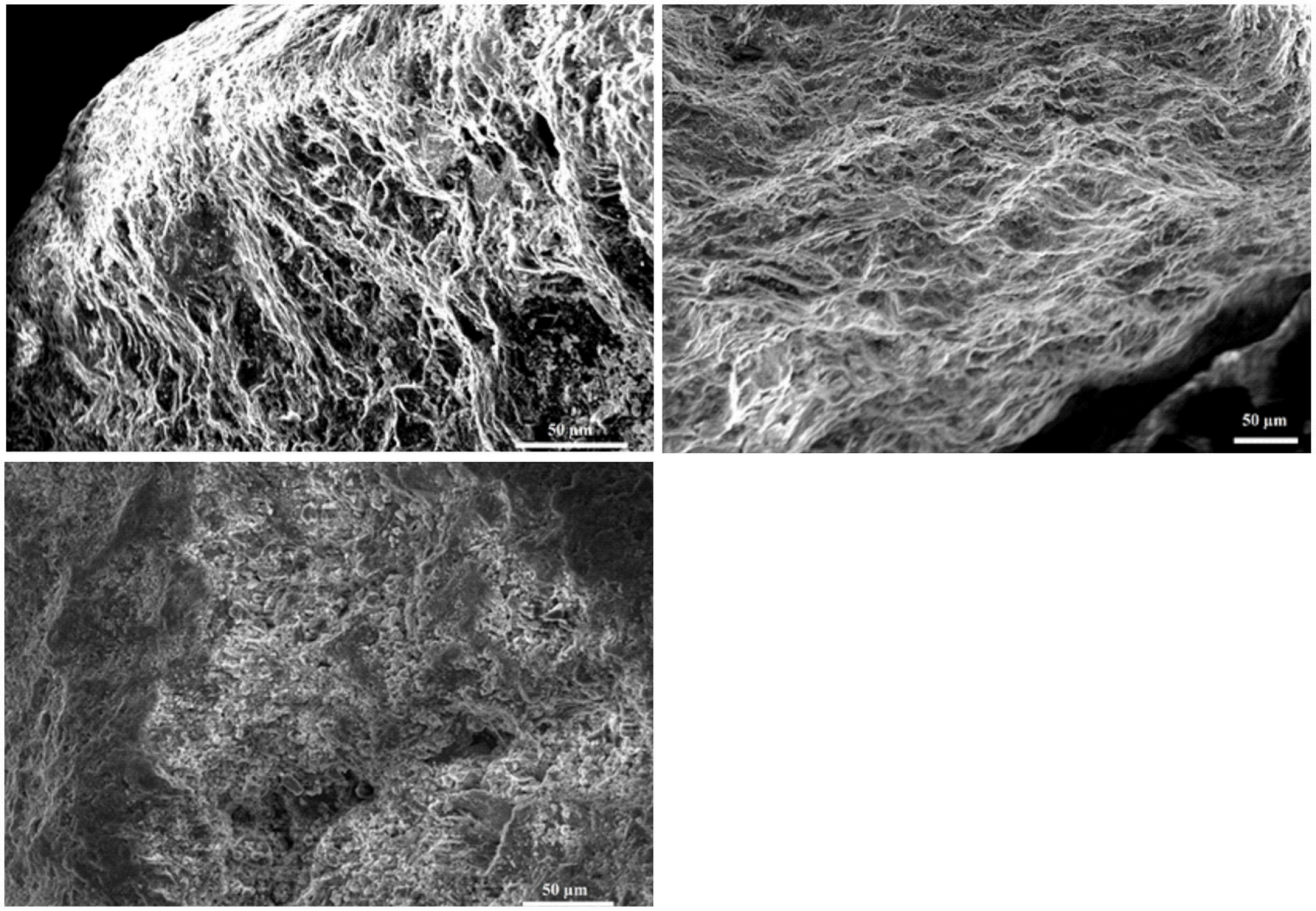
**Images obtained by SEM microscopy**. Upper left is a SEM image of basalt from the flight experiment and upper right a SEM image of basalt from the ground experiment. The control, lower right, is a SEM picture of the basalt after the 3 month experiment where no cells have been added and the surface of the basalt can be seen. Both in ground and flight experiment, biofilm covers the basalt surface. For the flight, less thick biofilm is seen as the surface is still visible and the biofilm does not cover the whole surface such as seen in the ground picture.

SEM analysis showed biofilm formation on basalt under flight conditions and in the ground control experiment, although the biofilm formed during flight conditions was less developed (Figure 3). On the ground the basalt surface is completely covered with biofilm cells, while for the flight samples some basalt surface can still be seen (Figure 3, flight). The level of biofilm formation in the ground control samples is comparable to that observed in preflight preparation experiments with the same setup (showed in Supplementary Figure S1).

#### Effect of space flight on cultivability of biofilm cells

**Figure 4:**
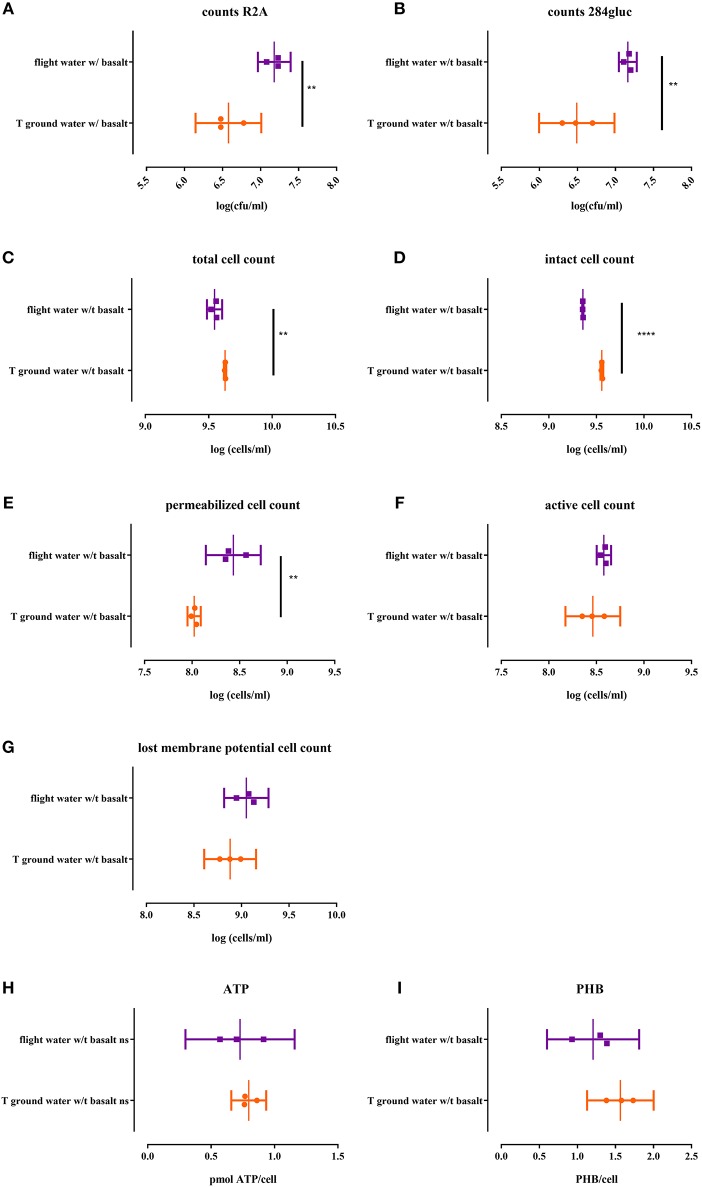
**Cell physiology, cultivability ATP and PHB levels of the CH34 biofilm fraction after sonication of both flight and ground samples with basalt, analyzed after the three month flight experiment**. All replicates are represented by a dot; the mean is indicated as a line as well as the 95% confidence interval between brackets. For the statistical analysis, one way ANOVA was used with Tukey post testing (alpha = 0.05). Significances are indicated with (^*^) (with: ^**^p < 0.01 and ^****^p < 0.0001). *P*-values as well as significance are also reported in Supplementary Table S2. Samples were plated on R2A **(A)** and 284 MM agar **(B)** to determine cultivability of CH34 biofilm cells. The total cells number (SG) **(C)** as well as the number of intact cells (SGPI) **(D)**, permeabilizated (SGPI) **(E)**, active (cFDA) **(G)** and cells which have lost their membrane potential [DIBAC_4_(3)] **(F)** were measured. ATP **(H)** and PHB **(I)** content of the cells was also measured.

When biofilm cells were cultured on R2A and 284 MM agar (Figures 4A,B respectively), the viable count for flight was significantly higher (*p* < 0.0058) than the ground samples. The number of cultivable cells in the biofilm fraction was also 1–1.4 log lower than in the planktonic cell fraction, indicating a significant impact (*p* < 0.0003) of the biofilm mode of growth on cultivability.

#### Effect of space flight on physiology of biofilm cells

The total biofilm cell number was significantly lower (*p* < 0.0004; Figure 4C) in the flight samples compared to the ground. Flight samples also contained significantly more permeabilizated cells (*p* > 0.004; Figure 4E) and less intact cells (*p* > 0.0001; Figure 4D). No significant differences were observed for activity (Figure 4G) and membrane potential (Figure 4F) between flight and ground conditions.

#### Effect of space flight on energy status of biofilm cells

No significant difference was observed between the intracellular ATP levels nor the PHB content of biofilm cells from the flight and ground experiment (Figure 4H). In contrast, the ATP content of the biofilm cells is 10x lower than the ATP content (*p* < 0.0006) of the planktonic cells while the intracellular PHB content does not significantly differ from the planktonic cells in suspension (Figure 4I).

### The effect of basalt on element release

**Figure 5:**
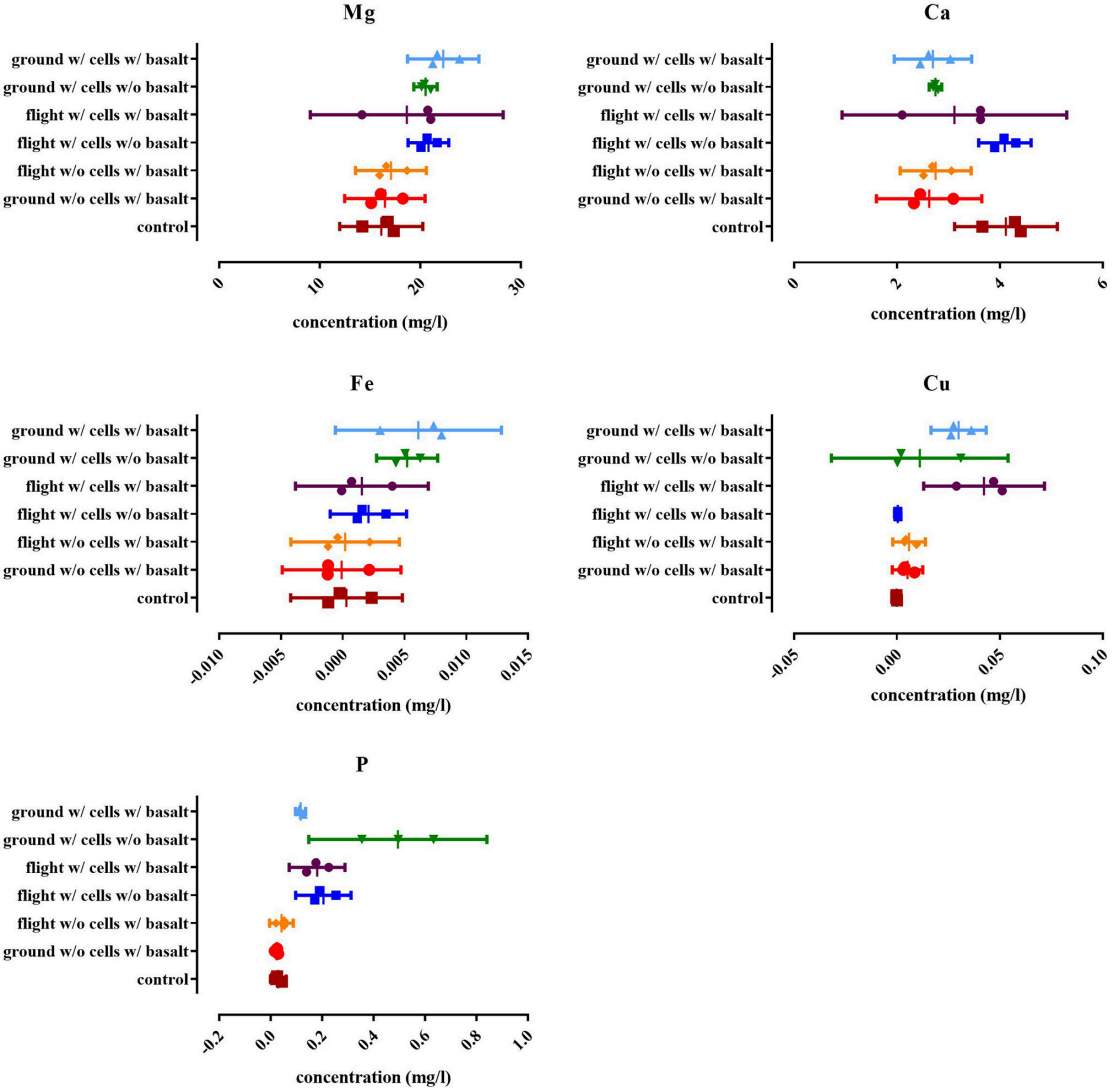
**Results of the ICP-OES analysis for the magnesium (Mg), total phosphate (P), calcium (Ca), iron (Fe), and copper (Cu) content expressed as the concentration in the suspension (in mg/l)**. The “control” is the concentration of the respective elements in the water used at the start of the experiment. All replicates are represented by a dot, the mean is indicated as well as the 95% confidence interval. For the statistical analysis, one way ANOVA was used with Tukey post testing (alpha = 0.05), *p*-values as well as significance are reported in Supplementary Table S2.

ICP-OES was performed to quantify magnesium, aluminum, calcium, iron, copper, and phosphate in solution and to evaluate the possible impact of CH34 cells on the leaching of elements from basalt (Figure 5). The long-term storage of basalt in mineral water did not significantly impact the concentration of any of the five tested elements, except for calcium. Water with basalt, both from flight and ground contained significantly less calcium compared to the start (*p* < 0.0225), indicating that basalt triggered a calcium complexation and removal from the water. The presence of CH34 cells impacted these concentrations: in the ground experiments there was more magnesium found in the water with cells with basalt compared to samples from the ground without cells with basalt (*p* < 0.0275) and the start of the experiment (*p* < 0.0181). No significant difference could be seen for magnesium in the flight samples compared to the ground samples. This is also the case for iron where samples from the ground samples contained significantly more iron compared to the condition without cells (*p* < 0.0254) and with and without basalt (*p* < 0.0468). Next, both ground and flight samples with basalt with cells contained significantly more copper than with basalt but without cells (*p* < 0.0013) and with cells but without basalt (*p* < 0.006). Flight and ground samples were however not significantly different from one another for copper (*p* > 0.5597). For calcium, only the cells from the flight experiment without basalt were significantly different from the other conditions (*p* < 0.0251), not showing any calcium complexation because basalt was not present in the samples. Phosphate concentrations in the ground and flight samples without basalt were significant higher than the conditions which contained basalt with (*p* < 0.0004) and without cells (*p* < 0.0491).

## Discussion

To evaluate the effect of space flight conditions on the survival of *C. metallidurans* CH34, a 3-month space flight experiment on board the Russian FOTON-M4 capsule was conducted with cells suspended in mineral water. In addition, the effect of the presence of basalt and biofilm formation on basalt was scrutinized.

As expected, long-term storage had a significantly detrimental effect on physiology and cultivability of CH34 cells. Both flight and ground samples with and without basalt had lower cultivability, less ATP and more PHB per cell compared to the beginning of the experiment, and in addition contained more permeabilizated cells and cells that have lost their membrane potential.

Decreased cultivability, but no decline in total cell numbers, indicates that the cells surviving in these oligotrophic conditions transition into a more dormant state. This is already been shown in other experiments in similar oligotrophic conditions (Kell et al., 1998; Oliver, 2005). Both the presence of basalt and flight conditions had a positive effect, lessening the impact of the survival conditions, where 10% of the culture was cultivable with basalt and 50% in flight in contrast to 0.1% in the ground experiment.

The energy status of these cells was also assessed by analyzing the ATP and PHB content, as in CH34 most of its stored energy is in the formation of polyhydroxybuterates (PHB) (Sato et al., 2004; Janssen et al., 2010; Budde et al., 2011). When ATP levels were compared to the start, these were significantly lower and PHB significantly increased compared to the starting conditions. The CH34 cells thus reduce their “immediate operational” energy levels (ATP) but increase their energy storage levels (PHB) when put in these survival conditions. Space flight conditions seem to counteract this decrease in ATP content, while basalt limited the PHB accumulation of the cells.

The drop in cultivability thus coincides with a 3-fold decrease in energy levels (measured ATP), and a 2-fold increase in energy stock levels (PHB) over the 3 months flight experiment. Previous studies show that cells accumulate PHB in nutrient poor environments in mineral water (Kadouri et al., 2005). Although, not investigated here, PHB accumulation could result from metabolic redirecting of proteome or lipid cellular fractions seen in closely related *Ralstonia eutropha* H12 (Brigham et al., 2010; Sharma et al., 2016); cryptic growth as more permeabilizated cells are seen in these conditions (McAlister et al., 2002); usage of leached byproducts of the plastic tubes (Jones et al., 2003) or residual organic fractions still present on the basalt. Other factors such as exposure to stress conditions (Povolo and Casella, 2000; Rojas et al., 2011) and phosphate limitation (Shang et al., 2003; Budde et al., 2011) can trigger PHB accumulation as well. It has also been shown that PHB utilization during starvation conditions results in metabolic activity and cultivability (James et al., 1999; Trevors, 2011; Najdegerami et al., 2012). PHB accumulation on the other hand can result in loss of cultivability which is also seen in our results (Holmquist and Kjelleberg, 1993).

Flight conditions and basalt had a positive impact on physiology, counteracting some of the detrimental storage effects. Cells in the flight conditions with basalt contained the highest amount of total, intact and active cells while fewer cells lost their membrane potential. In addition, samples containing basalt, both on ground and in flight, contained less cells which were permeabilizated or lost their cell membrane potential and had higher total and intact fractions. In summary, fewer cells lose their cell membrane potential, more are active and keep a higher ATP level and lower PHB level resulting in higher cultivability. It has been seen before, in other experiments, with actively growing cells in culture medium, that indeed space flight can have a significant impact on physiology, predominately due to microgravity effects. It was shown that bacteria have increased metabolic activity, higher biomass, and produce more secondary metabolites in these conditions allowing growth (Leys et al., 2004; Nickerson et al., 2004; Mastroleo et al., 2009; Taylor, 2015). For cells tested in survival conditions, *Ralstonia pickettii*, starting from a 10^5^ cells/ml in water, showed a higher cell break down and autolysis rate in simulated microgravity compared to normal gravity after 14 days in this survival setup (Baker and Leff, 2004).

Space flight conditions have an impact on the bacterial cell physiology, which in turn can have an impact on microbial rock weathering and biofilm formation to maximize survival. Biofilm formation was different in flight and ground conditions, observed using SEM pictures. A clear biofilm was formed in ground conditions but this is not the case in the flight experiment in which the biofilm was shown to be less developed and differently structured. When total cell numbers were determined there was also a significant difference between ground and flight samples indicating that in flight fewer cells were present in the biofilm. This contrary to some papers where biofilm formation increased in space flight conditions (McLean et al., 2001). Cells present in the biofilm during flight also show different physiology traits, as more were permeabilizated, less intact, but more cultivable. In biofilm cells, ATP content or PHB content did not differ between ground and flight samples. Flight thus impacts the number of cells which transition into the biofilm state which increases cultivability but more cells are permeabilizated and less are intact. It was also observed that cells in the biofilm had significant less PHB then cells in suspension, indicating that part of the PHB may have been directed toward biofilm formation. It was already reported that PHB utilization could promote colonization and biofilm formation in nutrient poor environments, as it promotes motility and viability of these cells (Tribelli and López, 2011).

For the elemental analysis, basalt had an effect on calcium removal from the water, irrespectively of the presence of cells and both in ground and flight experiments, probably due to complexation on basalt (Stockmann et al., 2011). Also when adding cells, calcium is removed from the water even in samples without basalt, indicating that cells can take up calcium in this condition. This was also observed for phosphate as concentrations were lower, both in ground and flight experiments, with basalt, while without basalt more phosphate was remaining in suspension. Cells released more magnesium and iron with and without basalt in the ground samples while water from the flight experiment contained less of these elements, showing that cells keep more of these elements intracellularly in these conditions, irrespective of the presence of basalt. Copper is released from the basalt in the presence of cells both in flight and ground, thus cells having a positive impact on copper release from basalt.

Our results indicate that in general flight conditions as well as basalt had a positive effect on survival, counteracting some of the detrimental effects of inoculum storage in water. Cells were more cultivable, contained more ATP and more cells were present which were intact and active while fewer cells lost their membrane potential. This changes cellular physiology. Cells will thereby not as easily transition into a more “dormant” state and start forming a biofilm on basalt. With this experiment, we provide for the first time results for the combined effect of space flight conditions and the presence of basalt on survival in water. We could also show that in space cells form slightly less biofilm and so that there is an impact of space flight conditions on microbe-mineral interactions. As these experiments were performed with limited amount of samples and ground and flight samples were prepared separately, batch specific changes may have occurred. It is clear however that this preliminary experiment is only the very first step and much more is still to be tested and learned. Results presented here are preliminary results and more (long lasting) experiments are needed to draw definite conclusions. Nevertheless, this research may hopefully open the door for future studies and potentially applications of microbe-mineral interactions in space and even on Earth.

## Author contributions

BB performed the analysis after flight except for ICP-OES and radiation sensor read-out. ICP-OES was performed by NN and CC. IC prepared the flight setup and prepared the package before send-off. OV provided the radiation sensors included in the flight setup as well as read-out and data analysis. VI was responsible for incorporating the flight setup within Russian spaceflight experiment “MIKROB.” BB, IC, OV, NN, VI, CC, RV, NB, and NL helped with data interpretation, scientific guidance, and preparation of the manuscript.

### Conflict of interest statement

The authors declare that the research was conducted in the absence of any commercial or financial relationships that could be construed as a potential conflict of interest.

## Supplementary material

Supplementary Figure S1 **Scanning Electron Microscopy (SEM) image of basalt after preflight preparatory experiment with the same setup as the flight experiment**.

Supplementary Table S1 **Wavelengths used for ICP-OES analysis**.

Supplementary Table S2 **Statistical analysis of the planktonic, biofilm fractions as well as for the ICP-OES analysis**.
