# Supplementary figures and images for: The Impact of Space Flight on Survival and Interaction of Cupriavidus metallidurans CH34 with Basalt, a Volcanic Moon Analog Rock
Source: Front Microbiol. 2017 Apr 28;8:671. doi: 10.3389/fmicb.2017.00671 (PMC5408026; doi:10.3389/fmicb.2017.00671)

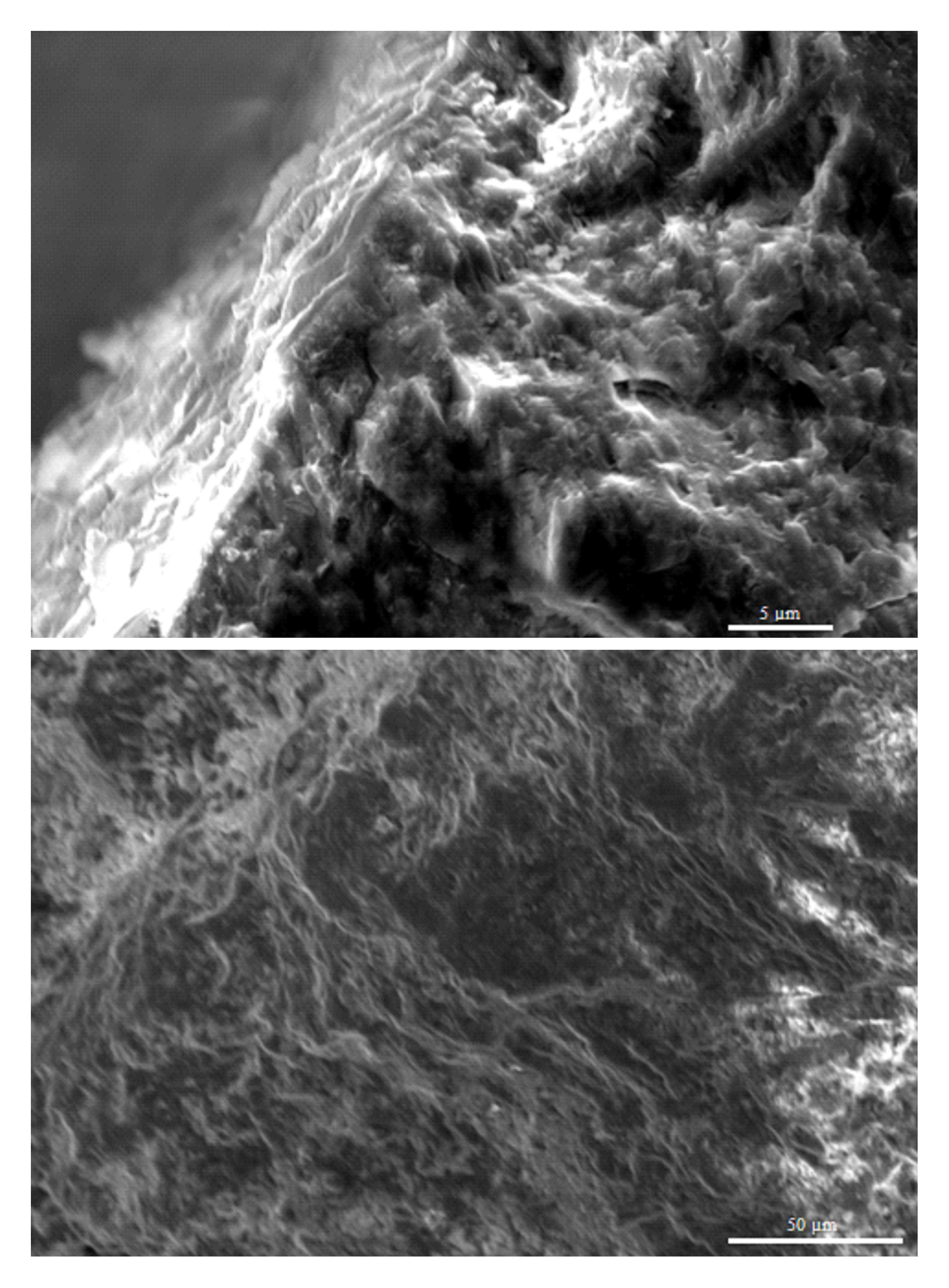

Supplement: Supplementary Figure S1 — Scanning Electron Microscopy (SEM) image of basalt after preflight preparatory experiment with the same setup as the flight experiment. [file Image1.TIF]
