# Supplementary material for: The Impact of Space Flight on Survival and Interaction of Cupriavidus metallidurans CH34 with Basalt, a Volcanic Moon Analog Rock
Source: Front Microbiol. 2017 Apr 28;8:671. doi: 10.3389/fmicb.2017.00671 (PMC5408026; doi:10.3389/fmicb.2017.00671)
Supplement: Supplementary Table S1 — Wavelengths used for ICP-OES analysis. [file Table1.DOCX]

Supplementary Material

The impact of space flight on survival and interaction of *Cupriavidus metallidurans* CH34 with basalt, a volcanic moon analog rock

**Bo Byloos^1,2^, Ilse Coninx^1^, Olivier Van Hoey^3^, Charles Cockell^4^, Natasha Nicholson^4^, Vyacheslav Ilyin^5^, Rob Van Houdt^1^, Nico Boon^2^ and Natalie Leys^1*^**

^1^Microbiology Unit, Belgian Nuclear Research Centre, SCK•CEN, Mol, Belgium.

^2^Center for Microbial Ecology and Technology (CMET), Ghent University, Gent, Belgium.

^3^Research in Dosimetric Applications, Belgian Nuclear Research Centre, SCK•CEN, Mol, Belgium.

^4^UK Centre for Astrobiology, School of Physics and Astronomy, University of Edinburgh, Edinburgh, United Kingdom.

^5^Institute of Medical and Biological Problems of Russian Academy of Sciences (IMBP RAS), Moscow, Russia.

*** Correspondance:** Dr. Natalie Leys, Natalie.Leys@sckcen.be

**Supplementary Table S1**: Wavelengths used for ICP-OES analysis

| Element | Wavelength |
| --- | --- |
| Al | 396.153 nm |
| Co | 228.616 nm |
| Cr | 205.568 nm |
| Cu | 324.752 nm |
| Fe | 259.945 nm |
| Mn | 257.61 nm |
| Ni | 221.656 nm |
| P | 214.914 nm |
| Ti | 336.121 nm |
| Zn | 206.205 nm |
| Ca | 317.933 nm |
| Mg | 280.271 nm |
| Si | 288.158 nm |
