# Supplementary material for: The Impact of Space Flight on Survival and Interaction of Cupriavidus metallidurans CH34 with Basalt, a Volcanic Moon Analog Rock
Source: Front Microbiol. 2017 Apr 28;8:671. doi: 10.3389/fmicb.2017.00671 (PMC5408026; doi:10.3389/fmicb.2017.00671)
Supplement: Supplementary Table S2 — Statistical analysis of the planktonic, biofilm fractions as well as for the ICP-OES analysis. [file Table2.DOCX]

Supplementary Material

The impact of space flight on survival and interaction of *Cupriavidus metallidurans* CH34 with basalt, a volcanic moon analog rock

**Bo Byloos^1,2^, Ilse Coninx^1^, Olivier Van Hoey^3^, Charles Cockell^4^, Natasha Nicholson^4^, Vyacheslav Ilyin^5^, Rob Van Houdt^1^, Nico Boon^2^ and Natalie Leys^1*^**

^1^Microbiology Unit, Belgian Nuclear Research Centre, SCK•CEN, Mol, Belgium.

^2^Center for Microbial Ecology and Technology (CMET), Ghent University, Gent, Belgium.

^3^Research in Dosimetric Applications, Belgian Nuclear Research Centre, SCK•CEN, Mol, Belgium.

^4^UK Centre for Astrobiology, School of Physics and Astronomy, University of Edinburgh, Edinburgh, United Kingdom.

^5^Institute of Medical and Biological Problems of Russian Academy of Sciences (IMBP RAS), Moscow, Russia.

*** Correspondance:** Dr. Natalie Leys, [Natalie.Leys@sckcen.be](mailto:Natalie.Leys@sckcen.be)

**Supplementary Table S2**: Statistical analysis of the planktonic, biofilm fractions as well as for the ICP-OES analysis.

| **Plantonic cell fraction** |  |  |  |  |  |
| --- | --- | --- | --- | --- | --- |
| Ordinary One way ANOVA with Turkey's post testing |  |  |  |  |  |
| Total |  |  |  |  |  |
|  | Mean Diff, | 95,00% CI of diff, | Significant? | Summary | Adjusted P Value |
| initial vs. T ground water w/o basalt | 0,1429 | 0,07843 to 0,2075 | Yes | *** | 0,0002 |
| initial vs. T ground water w/t basalt | 0,06952 | 0,005005 to 0,134 | Yes | * | 0,0336 |
| initial vs. flight water w/o basalt | 0,1466 | 0,0821 to 0,2111 | Yes | *** | 0,0002 |
| initial vs. flight water w/t basalt | -0,03168 | -0,0962 to 0,03284 | No | ns | 0,5201 |
| T ground water w/o basalt vs. T ground water w/t basalt | -0,07342 | -0,1379 to -0,008901 | Yes | * | 0,0247 |
| T ground water w/o basalt vs. flight water w/o basalt | 0,003676 | -0,06084 to 0,0682 | No | ns | 0,9997 |
| T ground water w/o basalt vs. flight water w/t basalt | -0,1746 | -0,2391 to -0,1101 | Yes | **** | <0,0001 |
| T ground water w/t basalt vs. flight water w/o basalt | 0,0771 | 0,01258 to 0,1416 | Yes | * | 0,0185 |
| T ground water w/t basalt vs. flight water w/t basalt | -0,1012 | -0,1657 to -0,03669 | Yes | ** | 0,003 |
| flight water w/o basalt vs. flight water w/t basalt | -0,1783 | -0,2428 to -0,1138 | Yes | **** | <0,0001 |
| Intact |  |  |  |  |  |
|  | Mean Diff, | 95,00% CI of diff, | Significant? | Summary | Adjusted P Value |
| initial vs. T ground water w/o basalt | 0,07804 | -0,003587 to 0,1597 | No | ns | 0,0626 |
| initial vs. T ground water w/t basalt | 0,05809 | -0,02353 to 0,1397 | No | ns | 0,2088 |
| initial vs. flight water w/o basalt | 0,3755 | 0,2938 to 0,4571 | Yes | **** | <0,0001 |
| initial vs. flight water w/t basalt | 0,0336 | -0,04802 to 0,1152 | No | ns | 0,6666 |
| T ground water w/o basalt vs. T ground water w/t basalt | -0,01994 | -0,1016 to 0,06168 | No | ns | 0,9235 |
| T ground water w/o basalt vs. flight water w/o basalt | 0,2974 | 0,2158 to 0,379 | Yes | **** | <0,0001 |
| T ground water w/o basalt vs. flight water w/t basalt | -0,04443 | -0,1261 to 0,03719 | No | ns | 0,4281 |
| T ground water w/t basalt vs. flight water w/o basalt | 0,3174 | 0,2357 to 0,399 | Yes | **** | <0,0001 |
| T ground water w/t basalt vs. flight water w/t basalt | -0,02449 | -0,1061 to 0,05713 | No | ns | 0,8551 |
| flight water w/o basalt vs. flight water w/t basalt | -0,3419 | -0,4235 to -0,2602 | Yes | **** | <0,0001 |
| Permeabilized |  |  |  |  |  |
|  | Mean Diff, | 95,00% CI of diff, | Significant? | Summary | Adjusted P Value |
| initial vs. T ground water w/o basalt | -1,191 | -1,426 to -0,9549 | Yes | **** | <0,0001 |
| initial vs. T ground water w/t basalt | -0,4086 | -0,6443 to -0,1728 | Yes | ** | 0,0014 |
| initial vs. flight water w/o basalt | -1,013 | -1,249 to -0,7776 | Yes | **** | <0,0001 |
| initial vs. flight water w/t basalt | -0,8678 | -1,103 to -0,632 | Yes | **** | <0,0001 |
| T ground water w/o basalt vs. T ground water w/t basalt | 0,7821 | 0,5464 to 1,018 | Yes | **** | <0,0001 |
| T ground water w/o basalt vs. flight water w/o basalt | 0,1773 | -0,05841 to 0,4131 | No | ns | 0,1724 |
| T ground water w/o basalt vs. flight water w/t basalt | 0,3229 | 0,08719 to 0,5587 | Yes | ** | 0,0078 |
| T ground water w/t basalt vs. flight water w/o basalt | -0,6048 | -0,8405 to -0,3691 | Yes | **** | <0,0001 |
| T ground water w/t basalt vs. flight water w/t basalt | -0,4592 | -0,6949 to -0,2235 | Yes | *** | 0,0006 |
| flight water w/o basalt vs. flight water w/t basalt | 0,1456 | -0,09013 to 0,3813 | No | ns | 0,318 |
| Active |  |  |  |  |  |
|  | Mean Diff, | 95,00% CI of diff, | Significant? | Summary | Adjusted P Value |
| initial vs. T ground water w/o basalt | 0,3692 | 0,2226 to 0,5157 | Yes | **** | <0,0001 |
| initial vs. T ground water w/t basalt | 0,5719 | 0,4254 to 0,7185 | Yes | **** | <0,0001 |
| initial vs. flight water w/o basalt | 0,3064 | 0,1599 to 0,4529 | Yes | *** | 0,0003 |
| initial vs. flight water w/t basalt | 0,1116 | -0,03491 to 0,2581 | No | ns | 0,1647 |
| T ground water w/o basalt vs. T ground water w/t basalt | 0,2028 | 0,05625 to 0,3493 | Yes | ** | 0,0073 |
| T ground water w/o basalt vs. flight water w/o basalt | -0,06275 | -0,2093 to 0,08378 | No | ns | 0,6358 |
| T ground water w/o basalt vs. flight water w/t basalt | -0,2575 | -0,4041 to -0,111 | Yes | ** | 0,0013 |
| T ground water w/t basalt vs. flight water w/o basalt | -0,2655 | -0,4121 to -0,119 | Yes | ** | 0,001 |
| T ground water w/t basalt vs. flight water w/t basalt | -0,4603 | -0,6068 to -0,3138 | Yes | **** | <0,0001 |
| flight water w/o basalt vs. flight water w/t basalt | -0,1948 | -0,3413 to -0,04826 | Yes | ** | 0,0095 |
| Lost membrane potential |  |  |  |  |  |
|  | Mean Diff, | 95,00% CI of diff, | Significant? | Summary | Adjusted P Value |
| initial vs. T ground water w/o basalt | -1,173 | -1,332 to -1,014 | Yes | **** | <0,0001 |
| initial vs. T ground water w/t basalt | -1,077 | -1,236 to -0,9182 | Yes | **** | <0,0001 |
| initial vs. flight water w/o basalt | -0,9928 | -1,152 to -0,8337 | Yes | **** | <0,0001 |
| initial vs. flight water w/t basalt | -0,7414 | -0,9005 to -0,5823 | Yes | **** | <0,0001 |
| T ground water w/o basalt vs. T ground water w/t basalt | 0,096 | -0,0631 to 0,2551 | No | ns | 0,3377 |
| T ground water w/o basalt vs. flight water w/o basalt | 0,1805 | 0,02144 to 0,3396 | Yes | * | 0,0251 |
| T ground water w/o basalt vs. flight water w/t basalt | 0,4319 | 0,2728 to 0,591 | Yes | **** | <0,0001 |
| T ground water w/t basalt vs. flight water w/o basalt | 0,08453 | -0,07456 to 0,2436 | No | ns | 0,4497 |
| T ground water w/t basalt vs. flight water w/t basalt | 0,3359 | 0,1768 to 0,495 | Yes | *** | 0,0003 |
| flight water w/o basalt vs. flight water w/t basalt | 0,2514 | 0,09231 to 0,4105 | Yes | ** | 0,0029 |
| R2A |  |  |  |  |  |
|  | Mean Diff, | 95,00% CI of diff, | Significant? | Summary | Adjusted P Value |
| initial vs. T ground water w/o basalt | 2,157 | 1,783 to 2,53 | Yes | **** | <0,0001 |
| initial vs. T ground water w/t basalt | 1,1 | 0,7263 to 1,474 | Yes | **** | <0,0001 |
| initial vs. flight water w/o basalt | 0,5 | 0,1263 to 0,8737 | Yes | ** | 0,0091 |
| initial vs. flight water w/t basalt | 0,4567 | 0,08297 to 0,8304 | Yes | * | 0,0162 |
| T ground water w/o basalt vs. T ground water w/t basalt | -1,057 | -1,43 to -0,683 | Yes | **** | <0,0001 |
| T ground water w/o basalt vs. flight water w/o basalt | -1,657 | -2,03 to -1,283 | Yes | **** | <0,0001 |
| T ground water w/o basalt vs. flight water w/t basalt | -1,7 | -2,074 to -1,326 | Yes | **** | <0,0001 |
| T ground water w/t basalt vs. flight water w/o basalt | -0,6 | -0,9737 to -0,2263 | Yes | ** | 0,0025 |
| T ground water w/t basalt vs. flight water w/t basalt | -0,6433 | -1,017 to -0,2696 | Yes | ** | 0,0015 |
| flight water w/o basalt vs. flight water w/t basalt | -0,04333 | -0,417 to 0,3304 | No | ns | 0,9947 |
| 284 |  |  |  |  |  |
|  | Mean Diff, | 95,00% CI of diff, | Significant? | Summary | Adjusted P Value |
| initial vs. T ground water w/o basalt | 2,44 | 2,071 to 2,809 | Yes | **** | <0,0001 |
| initial vs. T ground water w/t basalt | 1,11 | 0,7411 to 1,479 | Yes | **** | <0,0001 |
| initial vs. flight water w/o basalt | 0,6633 | 0,2944 to 1,032 | Yes | ** | 0,0011 |
| initial vs. flight water w/t basalt | 0,58 | 0,2111 to 0,9489 | Yes | ** | 0,003 |
| T ground water w/o basalt vs. T ground water w/t basalt | -1,33 | -1,699 to -0,9611 | Yes | **** | <0,0001 |
| T ground water w/o basalt vs. flight water w/o basalt | -1,777 | -2,146 to -1,408 | Yes | **** | <0,0001 |
| T ground water w/o basalt vs. flight water w/t basalt | -1,86 | -2,229 to -1,491 | Yes | **** | <0,0001 |
| T ground water w/t basalt vs. flight water w/o basalt | -0,4467 | -0,8156 to -0,07777 | Yes | * | 0,0171 |
| T ground water w/t basalt vs. flight water w/t basalt | -0,53 | -0,8989 to -0,1611 | Yes | ** | 0,0056 |
| flight water w/o basalt vs. flight water w/t basalt | -0,08333 | -0,4522 to 0,2856 | No | ns | 0,941 |
| ATP |  |  |  |  |  |
|  | Mean Diff, | 95,00% CI of diff, | Significant? | Summary | Adjusted P Value |
| initial vs. T ground water w/o basalt | 32,13 | 26,17 to 38,08 | Yes | **** | <0,0001 |
| initial vs. T ground water w/t basalt | 33,03 | 27,07 to 38,99 | Yes | **** | <0,0001 |
| initial vs. flight water w/o basalt | 23,68 | 17,72 to 29,64 | Yes | **** | <0,0001 |
| initial vs. flight water w/t basalt | 26,01 | 20,05 to 31,97 | Yes | **** | <0,0001 |
| T ground water w/o basalt vs. T ground water w/t basalt | 0,9038 | -5,056 to 6,863 | No | ns | 0,9856 |
| T ground water w/o basalt vs. flight water w/o basalt | -8,445 | -14,4 to -2,485 | Yes | ** | 0,0062 |
| T ground water w/o basalt vs. flight water w/t basalt | -6,111 | -12,07 to -0,1518 | Yes | * | 0,0439 |
| T ground water w/t basalt vs. flight water w/o basalt | -9,349 | -15,31 to -3,389 | Yes | ** | 0,003 |
| T ground water w/t basalt vs. flight water w/t basalt | -7,015 | -12,97 to -1,056 | Yes | * | 0,0203 |
| flight water w/o basalt vs. flight water w/t basalt | 2,334 | -3,626 to 8,293 | No | ns | 0,7036 |
| PHB |  |  |  |  |  |
|  | Mean Diff, | 95,00% CI of diff, | Significant? | Summary | Adjusted P Value |
| initial vs. T ground water w/o basalt | -1,679 | -2,296 to -1,061 | Yes | **** | <0,0001 |
| initial vs. T ground water w/t basalt | -0,9024 | -1,52 to -0,285 | Yes | ** | 0,005 |
| initial vs. flight water w/o basalt | -1,117 | -1,735 to -0,4998 | Yes | ** | 0,001 |
| initial vs. flight water w/t basalt | -1,027 | -1,644 to -0,4095 | Yes | ** | 0,002 |
| T ground water w/o basalt vs. T ground water w/t basalt | 0,7765 | 0,159 to 1,394 | Yes | * | 0,0135 |
| T ground water w/o basalt vs. flight water w/o basalt | 0,5617 | -0,0558 to 1,179 | No | ns | 0,0792 |
| T ground water w/o basalt vs. flight water w/t basalt | 0,6519 | 0,03446 to 1,269 | Yes | * | 0,0376 |
| T ground water w/t basalt vs. flight water w/o basalt | -0,2148 | -0,8323 to 0,4026 | No | ns | 0,7803 |
| T ground water w/t basalt vs. flight water w/t basalt | -0,1246 | -0,7421 to 0,4929 | No | ns | 0,9599 |
| flight water w/o basalt vs. flight water w/t basalt | 0,09026 | -0,5272 to 0,7077 | No | ns | 0,9874 |
|  |  |  |  |  |  |
| **Biofilm cell fraction** |  |  |  |  |  |
| Unpaired two tailed t-test |  |  |  |  |  |
|  | t, df | Significant? | Summary | P value |  |
| total | t=5,933 df=4 | Yes | ** | 0,0004 |  |
| intact cell count | t=51,22 df=4 | Yes | **** | <0,0001 |  |
| permeabilized cell count | t=5,96 df=4 | Yes | ** | 0,004 |  |
| active cell count | t=1,673 df=4 | No | ns | 0,1697 |  |
| lost membrane cell count | t=2,046 df=4 | No | ns | 0,1102 |  |
| R2A | t=5,374 df=4 | Yes | ** | 0,0058 |  |
| 284gluc | t=5,671 df=4 | Yes | ** | 0,0048 |  |
| PHB | t=2,067 df=4 | No | ns | 0,1076 |  |
| ATP | t=0,6559 df=4 | No | ns | 0,5477 |  |
|  |  |  |  |  |  |
| **ICP-OES** |  |  |  |  |  |
| Ordinary One way ANOVA with Turkey's post testing |  |  |  |  |  |
| Mg |  |  |  |  |  |
|  | Mean Diff, | 95,00% CI of diff, | Significant? | Summary | Adjusted P Value |
| flight w/ cells w/ basalt vs. flight w/ cells w/o basalt | -2,15 | -7,438 to 3,138 | No | ns | 0,7992 |
| flight w/ cells w/ basalt vs. ground w/ cells w/ basalt | -3,62 | -8,908 to 1,668 | No | ns | 0,2924 |
| flight w/ cells w/ basalt vs. ground w/ cells w/o basalt | -1,86 | -7,148 to 3,428 | No | ns | 0,8826 |
| flight w/ cells w/ basalt vs. flight w/o cells w/ basalt | 1,577 | -3,711 to 6,865 | No | ns | 0,9412 |
| flight w/ cells w/ basalt vs. ground w/o cells w/ basalt | 2,183 | -3,105 to 7,471 | No | ns | 0,7884 |
| flight w/ cells w/ basalt vs. control | 2,537 | -2,751 to 7,825 | No | ns | 0,6628 |
| flight w/ cells w/o basalt vs. ground w/ cells w/ basalt | -1,47 | -6,758 to 3,818 | No | ns | 0,9572 |
| flight w/ cells w/o basalt vs. ground w/ cells w/o basalt | 0,29 | -4,998 to 5,578 | No | ns | >0,9999 |
| flight w/ cells w/o basalt vs. flight w/o cells w/ basalt | 3,727 | -1,561 to 9,015 | No | ns | 0,2648 |
| flight w/ cells w/o basalt vs. ground w/o cells w/ basalt | 4,333 | -0,9545 to 9,621 | No | ns | 0,1441 |
| flight w/ cells w/o basalt vs. control | 4,687 | -0,6012 to 9,975 | No | ns | 0,0985 |
| ground w/ cells w/ basalt vs. ground w/ cells w/o basalt | 1,76 | -3,528 to 7,048 | No | ns | 0,906 |
| ground w/ cells w/ basalt vs. flight w/o cells w/ basalt | 5,197 | -0,09121 to 10,48 | No | ns | 0,0555 |
| ground w/ cells w/ basalt vs. ground w/o cells w/ basalt | 5,803 | 0,5155 to 11,09 | Yes | * | 0,0275 |
| ground w/ cells w/ basalt vs. control | 6,157 | 0,8688 to 11,44 | Yes | * | 0,0181 |
| ground w/ cells w/o basalt vs. flight w/o cells w/ basalt | 3,437 | -1,851 to 8,725 | No | ns | 0,3445 |
| ground w/ cells w/o basalt vs. ground w/o cells w/ basalt | 4,043 | -1,245 to 9,331 | No | ns | 0,1944 |
| ground w/ cells w/o basalt vs. control | 4,397 | -0,8912 to 9,685 | No | ns | 0,1348 |
| flight w/o cells w/ basalt vs. ground w/o cells w/ basalt | 0,6067 | -4,681 to 5,895 | No | ns | 0,9996 |
| flight w/o cells w/ basalt vs. control | 0,96 | -4,328 to 6,248 | No | ns | 0,9949 |
| ground w/o cells w/ basalt vs. control | 0,3533 | -4,935 to 5,641 | No | ns | >0,9999 |
| Fe |  |  |  |  |  |
|  | Mean Diff, | 95,00% CI of diff, | Significant? | Summary | Adjusted P Value |
| flight w/ cells w/ basalt vs. flight w/ cells w/o basalt | -0,000533 | -0,005772 to 0,004706 | No | ns | 0,9998 |
| flight w/ cells w/ basalt vs. ground w/ cells w/ basalt | -0,004575 | -0,009814 to 0,0006636 | No | ns | 0,1062 |
| flight w/ cells w/ basalt vs. ground w/ cells w/o basalt | -0,003661 | -0,0089 to 0,001578 | No | ns | 0,2727 |
| flight w/ cells w/ basalt vs. flight w/o cells w/ basalt | 0,001351 | -0,003887 to 0,00659 | No | ns | 0,9698 |
| flight w/ cells w/ basalt vs. ground w/o cells w/ basalt | 0,001635 | -0,003604 to 0,006873 | No | ns | 0,9284 |
| flight w/ cells w/ basalt vs. control | 0,00124 | -0,003999 to 0,006479 | No | ns | 0,9801 |
| flight w/ cells w/o basalt vs. ground w/ cells w/ basalt | -0,004042 | -0,009281 to 0,001197 | No | ns | 0,1873 |
| flight w/ cells w/o basalt vs. ground w/ cells w/o basalt | -0,003128 | -0,008367 to 0,002111 | No | ns | 0,4344 |
| flight w/ cells w/o basalt vs. flight w/o cells w/ basalt | 0,001884 | -0,003355 to 0,007123 | No | ns | 0,8719 |
| flight w/ cells w/o basalt vs. ground w/o cells w/ basalt | 0,002168 | -0,003071 to 0,007406 | No | ns | 0,7868 |
| flight w/ cells w/o basalt vs. control | 0,001773 | -0,003466 to 0,007012 | No | ns | 0,8994 |
| ground w/ cells w/ basalt vs. ground w/ cells w/o basalt | 0,000914 | -0,004325 to 0,006153 | No | ns | 0,9959 |
| ground w/ cells w/ basalt vs. flight w/o cells w/ basalt | 0,005926 | 0,0006875 to 0,01116 | Yes | * | 0,0223 |
| ground w/ cells w/ basalt vs. ground w/o cells w/ basalt | 0,00621 | 0,0009711 to 0,01145 | Yes | * | 0,0159 |
| ground w/ cells w/ basalt vs. control | 0,005815 | 0,0005764 to 0,01105 | Yes | * | 0,0254 |
| ground w/ cells w/o basalt vs. flight w/o cells w/ basalt | 0,005012 | -0,0002265 to 0,01025 | No | ns | 0,0649 |
| ground w/ cells w/o basalt vs. ground w/o cells w/ basalt | 0,005296 | 5,705e-005 to 0,01053 | Yes | * | 0,0468 |
| ground w/ cells w/o basalt vs. control | 0,004901 | -0,0003376 to 0,01014 | No | ns | 0,0737 |
| flight w/o cells w/ basalt vs. ground w/o cells w/ basalt | 0,0002836 | -0,004955 to 0,005522 | No | ns | >0,9999 |
| flight w/o cells w/ basalt vs. control | -0,0001111 | -0,00535 to 0,005128 | No | ns | >0,9999 |
| ground w/o cells w/ basalt vs. control | -0,0003947 | -0,005633 to 0,004844 | No | ns | >0,9999 |
| Ca |  |  |  |  |  |
|  | Mean Diff, | 95,00% CI of diff, | Significant? | Summary | Adjusted P Value |
| flight w/ cells w/ basalt vs. flight w/ cells w/o basalt | -0,98 | -2,188 to 0,2284 | No | ns | 0,1511 |
| flight w/ cells w/ basalt vs. ground w/ cells w/ basalt | 0,417 | -0,7914 to 1,625 | No | ns | 0,8912 |
| flight w/ cells w/ basalt vs. ground w/ cells w/o basalt | 0,3683 | -0,8401 to 1,577 | No | ns | 0,9353 |
| flight w/ cells w/ basalt vs. flight w/o cells w/ basalt | 0,3637 | -0,8448 to 1,572 | No | ns | 0,9388 |
| flight w/ cells w/ basalt vs. ground w/o cells w/ basalt | 0,4913 | -0,7171 to 1,7 | No | ns | 0,7993 |
| flight w/ cells w/ basalt vs. control | -1,001 | -2,21 to 0,2071 | No | ns | 0,137 |
| flight w/ cells w/o basalt vs. ground w/ cells w/ basalt | 1,397 | 0,1886 to 2,605 | Yes | * | 0,0191 |
| flight w/ cells w/o basalt vs. ground w/ cells w/o basalt | 1,348 | 0,1399 to 2,557 | Yes | * | 0,0245 |
| flight w/ cells w/o basalt vs. flight w/o cells w/ basalt | 1,344 | 0,1352 to 2,552 | Yes | * | 0,0251 |
| flight w/ cells w/o basalt vs. ground w/o cells w/ basalt | 1,471 | 0,2629 to 2,68 | Yes | * | 0,013 |
| flight w/ cells w/o basalt vs. control | -0,02133 | -1,23 to 1,187 | No | ns | >0,9999 |
| ground w/ cells w/ basalt vs. ground w/ cells w/o basalt | -0,04867 | -1,257 to 1,16 | No | ns | >0,9999 |
| ground w/ cells w/ basalt vs. flight w/o cells w/ basalt | -0,05333 | -1,262 to 1,155 | No | ns | >0,9999 |
| ground w/ cells w/ basalt vs. ground w/o cells w/ basalt | 0,07433 | -1,134 to 1,283 | No | ns | >0,9999 |
| ground w/ cells w/ basalt vs. control | -1,418 | -2,627 to -0,2099 | Yes | * | 0,0171 |
| ground w/ cells w/o basalt vs. flight w/o cells w/ basalt | -0,004667 | -1,213 to 1,204 | No | ns | >0,9999 |
| ground w/ cells w/o basalt vs. ground w/o cells w/ basalt | 0,123 | -1,085 to 1,331 | No | ns | 0,9998 |
| ground w/ cells w/o basalt vs. control | -1,37 | -2,578 to -0,1612 | Yes | * | 0,022 |
| flight w/o cells w/ basalt vs. ground w/o cells w/ basalt | 0,1277 | -1,081 to 1,336 | No | ns | 0,9998 |
| flight w/o cells w/ basalt vs. control | -1,365 | -2,573 to -0,1566 | Yes | * | 0,0225 |
| ground w/o cells w/ basalt vs. control | -1,493 | -2,701 to -0,2842 | Yes | * | 0,0117 |
| Cu |  |  |  |  |  |
|  | Mean Diff, | 95,00% CI of diff, | Significant? | Summary | Adjusted P Value |
| flight w/ cells w/ basalt vs. flight w/ cells w/o basalt | 0,0419 | 0,01867 to 0,06513 | Yes | *** | 0,0004 |
| flight w/ cells w/ basalt vs. ground w/ cells w/ basalt | 0,01235 | -0,01088 to 0,03558 | No | ns | 0,5597 |
| flight w/ cells w/ basalt vs. ground w/ cells w/o basalt | 0,0312 | 0,007974 to 0,05443 | Yes | ** | 0,006 |
| flight w/ cells w/ basalt vs. flight w/o cells w/ basalt | 0,03642 | 0,01319 to 0,05965 | Yes | ** | 0,0015 |
| flight w/ cells w/ basalt vs. ground w/o cells w/ basalt | 0,03719 | 0,01396 to 0,06042 | Yes | ** | 0,0013 |
| flight w/ cells w/ basalt vs. control | 0,04245 | 0,01922 to 0,06568 | Yes | *** | 0,0003 |
| flight w/ cells w/o basalt vs. ground w/ cells w/ basalt | -0,02955 | -0,05278 to -0,006324 | Yes | ** | 0,0093 |
| flight w/ cells w/o basalt vs. ground w/ cells w/o basalt | -0,0107 | -0,03393 to 0,01253 | No | ns | 0,7004 |
| flight w/ cells w/o basalt vs. flight w/o cells w/ basalt | -0,005481 | -0,02871 to 0,01775 | No | ns | 0,9804 |
| flight w/ cells w/o basalt vs. ground w/o cells w/ basalt | -0,004712 | -0,02794 to 0,01852 | No | ns | 0,9909 |
| flight w/ cells w/o basalt vs. control | 0,0005515 | -0,02268 to 0,02378 | No | ns | >0,9999 |
| ground w/ cells w/ basalt vs. ground w/ cells w/o basalt | 0,01886 | -0,004373 to 0,04209 | No | ns | 0,1505 |
| ground w/ cells w/ basalt vs. flight w/o cells w/ basalt | 0,02407 | 0,0008432 to 0,0473 | Yes | * | 0,0401 |
| ground w/ cells w/ basalt vs. ground w/o cells w/ basalt | 0,02484 | 0,001612 to 0,04807 | Yes | * | 0,0327 |
| ground w/ cells w/ basalt vs. control | 0,0301 | 0,006876 to 0,05333 | Yes | ** | 0,008 |
| ground w/ cells w/o basalt vs. flight w/o cells w/ basalt | 0,005216 | -0,01801 to 0,02845 | No | ns | 0,9847 |
| ground w/ cells w/o basalt vs. ground w/o cells w/ basalt | 0,005985 | -0,01724 to 0,02921 | No | ns | 0,97 |
| ground w/ cells w/o basalt vs. control | 0,01125 | -0,01198 to 0,03448 | No | ns | 0,6539 |
| flight w/o cells w/ basalt vs. ground w/o cells w/ basalt | 0,0007687 | -0,02246 to 0,024 | No | ns | >0,9999 |
| flight w/o cells w/ basalt vs. control | 0,006032 | -0,0172 to 0,02926 | No | ns | 0,9688 |
| ground w/o cells w/ basalt vs. control | 0,005264 | -0,01797 to 0,02849 | No | ns | 0,984 |
| P |  |  |  |  |  |
|  | Mean Diff, | 95,00% CI of diff, | Significant? | Summary | Adjusted P Value |
| flight w/ cells w/ basalt vs. flight w/ cells w/o basalt | -0,0244 | -0,1869 to 0,1381 | No | ns | 0,9982 |
| flight w/ cells w/ basalt vs. ground w/ cells w/ basalt | 0,06397 | -0,09855 to 0,2265 | No | ns | 0,8208 |
| flight w/ cells w/ basalt vs. ground w/ cells w/o basalt | -0,3138 | -0,4763 to -0,1513 | Yes | *** | 0,0002 |
| flight w/ cells w/ basalt vs. flight w/o cells w/ basalt | 0,1386 | -0,02393 to 0,3011 | No | ns | 0,1195 |
| flight w/ cells w/ basalt vs. ground w/o cells w/ basalt | 0,1569 | -0,005658 to 0,3194 | No | ns | 0,0617 |
| flight w/ cells w/ basalt vs. control | 0,1508 | -0,01175 to 0,3133 | No | ns | 0,0772 |
| flight w/ cells w/o basalt vs. ground w/ cells w/ basalt | 0,08837 | -0,07415 to 0,2509 | No | ns | 0,5355 |
| flight w/ cells w/o basalt vs. ground w/ cells w/o basalt | -0,2894 | -0,4519 to -0,1269 | Yes | *** | 0,0004 |
| flight w/ cells w/o basalt vs. flight w/o cells w/ basalt | 0,163 | 0,0004683 to 0,3255 | Yes | * | 0,0491 |
| flight w/ cells w/o basalt vs. ground w/o cells w/ basalt | 0,1813 | 0,01874 to 0,3438 | Yes | * | 0,0246 |
| flight w/ cells w/o basalt vs. control | 0,1752 | 0,01265 to 0,3377 | Yes | * | 0,031 |
| ground w/ cells w/ basalt vs. ground w/ cells w/o basalt | -0,3777 | -0,5402 to -0,2152 | Yes | **** | <0,0001 |
| ground w/ cells w/ basalt vs. flight w/o cells w/ basalt | 0,07462 | -0,0879 to 0,2371 | No | ns | 0,7031 |
| ground w/ cells w/ basalt vs. ground w/o cells w/ basalt | 0,09289 | -0,06963 to 0,2554 | No | ns | 0,4818 |
| ground w/ cells w/ basalt vs. control | 0,0868 | -0,07572 to 0,2493 | No | ns | 0,5546 |
| ground w/ cells w/o basalt vs. flight w/o cells w/ basalt | 0,4524 | 0,2898 to 0,6149 | Yes | **** | <0,0001 |
| ground w/ cells w/o basalt vs. ground w/o cells w/ basalt | 0,4706 | 0,3081 to 0,6331 | Yes | **** | <0,0001 |
| ground w/ cells w/o basalt vs. control | 0,4645 | 0,302 to 0,627 | Yes | **** | <0,0001 |
| flight w/o cells w/ basalt vs. ground w/o cells w/ basalt | 0,01827 | -0,1442 to 0,1808 | No | ns | 0,9996 |
| flight w/o cells w/ basalt vs. control | 0,01218 | -0,1503 to 0,1747 | No | ns | >0,9999 |
| ground w/o cells w/ basalt vs. control | -0,006093 | -0,1686 to 0,1564 | No | ns | >0,9999 |
